# Olfactory receptor 2 activation in macrophages: novel mediator of atherosclerosis progression

**DOI:** 10.1038/s41392-022-01115-7

**Published:** 2022-07-21

**Authors:** Zuowen He, Dao Wen Wang

**Affiliations:** 1 Division of Cardiology, Department of Internal Medicine, Tongji Hospital, Tongji Medical College, Huazhong University of Science and Technology, 430030 Wuhan, China; 2 Hubei Key Laboratory of Genetics and Molecular Mechanism of Cardiological Disorders, 430030 Wuhan, China

**Keywords:** Cardiology, Physiology

In a recent study published in *Science*, Orecchioni et al. provide insights that olfactory receptor 2 (OLFR2) in vascular macrophages plays a substantial role during atherosclerosis formation, identifying an alternative target for the prevention and treatment of ASCVD.^1^

Hypercholesterolemia and high low-density lipoprotein (LDL) levels have been identified as a major risk factor of atherosclerotic cardiovascular disease (ASCVD), the leading cause of death in humans. LDL-lowering therapies effectively reduce ASCVD risk by ~30–50%^2^ and are recommended for primary and secondary prevention. However, a considerable number of patients with ASCVD show poor responses to LDL-lowering treatment; therefore, identifying and controlling additional risk factors are key to the reduction of ASCVD morbidity.

Olfactory receptors (ORs) belong to a structurally diverse family of proteins capable of detecting a large spectrum of odorants in the environment. In vertebrates, including humans, ORs are typically located on the surface of sensory neurons in the olfactory epithelium. However, ORs also occur in tissues outside the olfactory epithelium. Parmentier et al. first identified OR gene transcripts expressed outside the olfactory epithelium in mammalian germ cells.^3^ Subsequent studies have shown that ORs are also located in other human tissues, including the lungs, testis, intestines, heart, skin, and blood.^4^ Although ORs outside the olfactory system are thought to participate in several essential physiological and pathophysiological processes, including pathfinding, cell growth, migration, secretion, differentiation, and apoptosis,^4^ understanding of their functions in the cardiovascular system remains poor. Ley and colleagues unexpectedly found a high expression of ORs in mouse macrophages from the atherosclerotic aorta in a transcriptomic study.^5^ They extended these findings and confirmed that OLFR2 was expressed in macrophages derived from atherosclerotic aorta and bone marrow, with specific biological functions. Octanal, an important OLFR2 ligand, is generated by lipid peroxidation and has been detected in oxidized low-density lipoprotein (oxLDL), which is one of the most important risk factors for atherosclerosis. These results suggest that octanal and OLFR2 are associated with atherosclerosis, encouraging the authors to investigate the function and regulation of OLFR2, and especially its relevance to atherosclerosis.

To investigate the downstream function of OLFR2, the authors performed RNA sequencing and pathway analysis and found that the most pronounced pathway altered in response to octanal stimulation was the oxidative stress pathway. This was further confirmed by MitoSox and dihydrorhodamine staining. Given the fact that reactive oxygen species (ROS) trigger signal 2 of the NLR family pyrin domain-containing 3 (NLRP3) inflammasome and subsequent release of interleukin-1β (IL-1β), the authors explored the impact of OLFR2 on the NLRP3 inflammasome pathway. Results showed that the secretion of inflammatory cytokine IL-1β and LDH (lactate dehydrogenase) were significantly induced by octanal in LPS (lipopolysaccharide)-primed mouse macrophages. This effect was abrogated by pharmacological inhibition or genetic deletion of OLFR2, NLRP3, and their downstream components. These results demonstrated that OLFR2 ligation increased the release of IL-1β by activating the NLRP3 inflammasome. Interestingly, treatment with octanal alone (without LPS) promoted the release of IL-1β by macrophages from the atherosclerotic aorta, suggesting that there may remain endogenous ligands of Toll-like receptor 4 (TLR4) that initiate the priming signal and permit the octanal to induce the release of IL-1β, IL-1α, and LDH. This raises the possibility that atherogenic ligands or pathogen-associated molecules may serve as priming signals for inflammasome activation in vascular macrophages. However, the spectrum of endogenous atherogenic ligands has not yet been fully elucidated.

As aforementioned that octanal binding to OLFR2 triggers NLRP3 inflammasome activation and subsequent IL-1β release, which have been previously identified to play a central role in the progression of atherosclerosis. Orecchioni et al. further explored the causative relationship of the OLFR2 activation with atherosclerosis. First, they demonstrated that octanal was significantly elevated in the plasma of atherosclerotic mice and can be generated by oleic acid in situ in the aorta at a much higher level than in the plasma. Meanwhile, octanal was detectable in human samples at levels comparable to those in mice and was positively correlated with triglycerides, total cholesterol, LDL, and non-high-density lipoprotein cholesterol. These results demonstrate a relationship between octanal and atherosclerosis in both mice and humans. This research team then tested the direct effect of octanal in a mouse model of atherosclerosis and found that supplementation with octanal aggravated atherosclerosis, as evidenced by the larger plaque lesion size and higher plasma levels of inflammatory cytokines. Genetic deletion of OLFR2, receptor transporter proteins 1 and 2, and downstream adenylate cyclase 3 in the bone marrow ameliorated atherosclerosis, as evidenced by a reduction in plaque vulnerability and an increase in collagen content, which are related to plaque stability. These results reveal the potent role of macrophage OLFR2 in the development of atherosclerosis. Importantly, this study also confirmed that OR6A2, the human ortholog of OLFR2, is also expressed in macrophages of the atherosclerotic aorta and has a similar function as OLFR2, not only in triggering Ca^2+^ flux but also in activating the NLRP3 inflammasome, suggesting that the mechanisms demonstrated in the mouse model are applicable to human disease.

Orecchioni et al. provided solid evidence that OLFR2 and its human ortholog, OR6A2, are expressed outside the olfactory epithelium in vascular macrophages. Although the functions of several olfactory receptors expressed outside the olfactory epithelium have been identified, the present study unexpectedly discovered the crucial role of OLFR2 and its human ortholog OR6A2 in atherosclerosis progression. Moreover, the plasma and aorta of atherosclerotic mice exhibited elevated levels of octanal, whose major sources are not food or gut microbiota products. Therefore, it is rational to propose that increased levels of octanal in the aorta and plasma may represent a response to factors that elicit atherosclerosis and could therefore act as a biomarker for atherosclerosis. Interestingly, the expression of OLFR2 was increased after treatment with the TLR4 agonist LPS and further enhanced in the presence of octanal. The possible mechanism is that LPS activates TLR4 and subsequently promotes nuclear translocation of NF-κB and AP-1, which may initiate the expression of OLFR2. This could represent possible effects of gut microbiota dysregulation, which facilitates passage of LPS from intestinal barriers and thus promotes inflammatory-related diseases, such as atherosclerosis and type 2 diabetes mellitus (T2DM). Mechanistically, this study further elucidated the crosstalk between lipids and inflammation remaining in macrophages, in which the NLRP3 inflammasome and its downstream IL-1β act as executors to initiate the inflammatory response that ultimately leads to atherosclerosis. Given that OR6A2 lies upstream of the NLRP3 inflammasome, OR6A2 could serve as a potential therapeutic target for inflammation initiated by lipid, especially, for the prevention, treatment, and reversal of atherosclerosis. For example, OR6A2 inhibitor combining with lipid-lowering drugs could have more potent effects in atherosclerosis therapy than using lipid-lowering drugs alone. Moreover, the anti-inflammatory effects of lipid-lowering drugs may partly depend on suppressing the octanal release and subsequent OR6A2 activation. Beyond OR6A2, other human olfactory receptors may also exert similar functions and potentially serve as therapeutical targets. However, challenges regarding drug discovery by intervening olfactory receptors may remain because of the potential side effects of such a drug that may also block or inhibit the olfaction.

Some key issues are worth further consideration. Although the role of octanal in triggering OLFR2 and its downstream events has been elegantly defined, it is unclear whether other olfactory receptors also participate in the development of atherosclerosis. Beyond octanal, is there any other medium-chain aliphatic aldehyde that initiates the OLFR2 signal and its mediated biological events, such as atherosclerosis? What is the major source of octanal produced in situ in atherosclerotic aorta? Is it mainly derived from the oxLDL? Since OR6A2, in conjunction with TLR4 ligation, induces inflammasome activation, does OR6A2 interact with TLR4 or they form heterodimer structurally? Given that lipid-driven inflammation is one of the key mechanisms of atherosclerosis, it is possible that macrophage OLFR2 activation may has a similar pathophysiological role in other diseases associated with lipid-driven inflammation, such as diabetes, obesity, cancer, neurodegeneration, and heart failure with preserved ejection fraction. Because some olfactory receptors are located outside the olfactory system, the expression and function of other types of sensory receptors, such as photoreceptors or taste receptors, outside of their typical locations should be investigated.

Taken together, these findings demonstrated that OLFR2 ligation aggravates atherosclerosis progression by triggering NLRP3 inflammasome activation and subsequent proinflammatory IL-1β release (Fig. 1). This study is the first to reveal the interplay between olfactory receptors and the pathological process of atherosclerosis, providing new biomarkers and therapeutic targets for ASCVD.

**Fig. 1:**
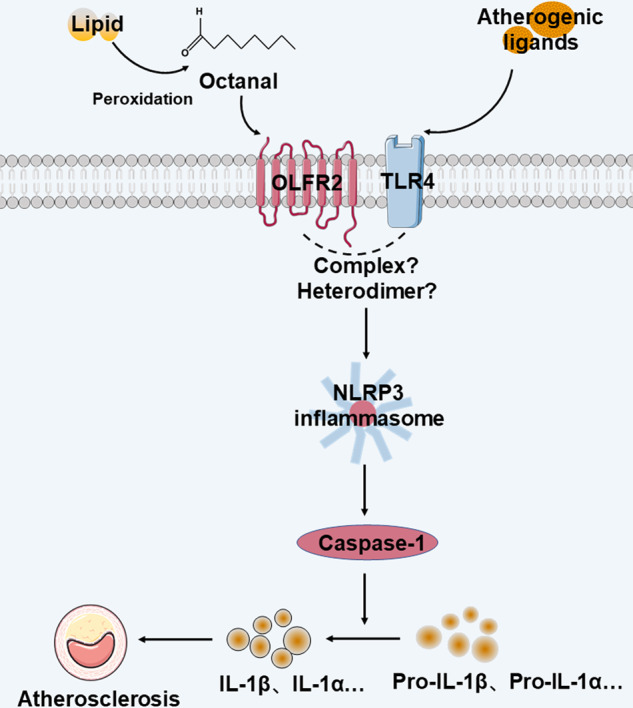
Schematic showing mechanisms of OLFR2 ligation-mediated signaling promoting atherosclerosis. Endogenous atherogenic ligands activate TLR4 and subsequently initiate the priming signal of the NLRP3 inflammasome. Octanal derived from oxLDL activates the OLFR2 and the downstream NLRP3 inflammasome activation, which triggers inflammatory cytokine interleukin release, leading to the progression of atherosclerosis. OLFR2 olfactory receptor 2, TLR4 Toll-like receptor 4, NLRP3 NLR family pyrin domain-containing 3, oxLDL oxidized low-density lipoprotein, IL-1β interleukin-1β, IL-1α interleukin-1α. This figure was designed using smart.servier.com

## References

[1] Orecchioni M (2022). Olfactory receptor 2 in vascular macrophages drives atherosclerosis by NLRP3-dependent IL-1 production. Science.

[2] Cholesterol Treatment Trialists, C. (2010). Efficacy and safety of more intensive lowering of LDL cholesterol: a meta-analysis of data from 170,000 participants in 26 randomised trials. Lancet.

[3] Parmentier M (1992). Expression of members of the putative olfactory receptor gene family in mammalian germ cells. Nature.

[4] Massberg D, Hatt H (2018). Human olfactory receptors: novel cellular functions outside of the nose. Physiol. Rev..

[5] McArdle S (2019). Migratory and dancing macrophage subsets in atherosclerotic lesions. Circulation Res..

